# Hemodialysis patients’ preferences for the management of secondary hyperparathyroidism

**DOI:** 10.1186/s12882-017-0665-8

**Published:** 2017-07-28

**Authors:** Brett Hauber, John Caloyeras, Joshua Posner, Deborah Brommage, Vasily Belozeroff, Kerry Cooper

**Affiliations:** 1 RTI Health Solutions, Offices Drive, Research Triangle Park, Park, NC 200 USA; 2 Amgen, Thousand Oaks, CA USA; 3 National Kidney Foundation, New York, NY USA

**Keywords:** Secondary hyperparathyroidism, End-stage renal disease, Discrete-choice experiment, Conjoint analysis

## Abstract

**Background:**

Patient engagement and patient-centered care are critical in optimally managing patients with end-stage renal disease (ESRD). Understanding patient preferences is a key element of patient-centered care and shared decision making. The objective of this study was to elicit patients’ preferences for the treatment of secondary hyperparathyroidism (SHPT) associated with ESRD using a discrete-choice experiment survey.

**Methods:**

Clinical literature, nephrologist input, patient-education resources, and a patient focus group informed development of the survey instrument, which was qualitatively pretested before its administration to a broader sample of patients. The National Kidney Foundation invited individuals in the United States with ESRD who were undergoing hemodialysis to participate in the survey. Respondents chose among three hypothetical SHPT treatment alternatives (two medical alternatives and surgery) in each of a series of questions, which were defined by attributes of efficacy (effect on laboratory values and symptoms), safety, tolerability, mode of administration, and cost. The survey instrument included a best-worst scaling exercise to quantify the relative bother of the individual attributes of surgery. Random-parameters logit models were used to evaluate the conditional relative importance of the attributes.

**Results:**

A total of 200 patients with ESRD completed the survey. The treatment attributes that were most important to the respondents were whether a treatment was a medication or surgery and out-of-pocket cost. Patients had statistically significant preferences for efficacy attributes related to symptom management and laboratory values, but placed less importance on the attributes related to mode of administration and side effects. The most bothersome attribute of surgery was the risk of surgical mortality.

**Conclusions:**

Patients with ESRD and SHPT who are undergoing hemodialysis understand SHPT and have clear and measurable treatment preferences. These results may help inform clinicians about patients’ preferences regarding treatment options for a common complication of ESRD.

**Electronic supplementary material:**

The online version of this article (doi:10.1186/s12882-017-0665-8) contains supplementary material, which is available to authorized users.

## Background

Patient engagement and patient-centered care are critical to the optimal management of end-stage renal disease (ESRD) and may improve patients’ outcomes and satisfaction with treatment [1, 2]. The “pinnacle” of patient-centered care is shared decision making, whereby patients and physicians agree on a treatment strategy that accounts for the benefits and risks of the available options and patients’ priorities for treatment [3]. To promote shared decision making in ESRD, patients’ goals, values, and preferences must be elicited, and treatment strategies should be tailored to reflect what is most important to patients [1]. However, owing to the clinical complexity of ESRD, as well as limited time and competing educational priorities for providers, it may be difficult for a provider to fully understand a patient’s preferences for each of the many treatment decisions that must be made in ESRD.

One example of a complication of ESRD for which treatment decisions must be made is secondary hyperparathyroidism (SHPT), which is characterized by imbalances in bone and mineral metabolism due to diminished kidney function and particularly manifests in elevated blood levels of parathyroid hormone. SHPT is estimated to affect 72% of patients with stage 4 or 5 chronic kidney disease [4]. The clinical consequences of SHPT include increased risks of bone disease and vascular calcification [5–7].

Treatment options for SHPT center on medications (e.g., vitamin D; oral cinacalcet; and intravenous etelcalcetide, an investigational agent) and surgery (parathyroidectomy). Beyond differences in the type and mode of intervention, each treatment has its own benefit and risk profile, resulting in potentially complex treatment decisions for providers and patients in the management of SHPT. To inform a shared decision-making approach in the management of ESRD and its complications, we sought to elicit patients’ preferences for SHPT treatment using a discrete-choice experiment (DCE) survey. A study using similar methods was conducted to evaluate patients’ preferences for the management of anemia [8].

## Methods

DCEs ask patients to choose among hypothetical treatment options defined by attributes that can take on different levels (see Table 1). By analyzing the pattern of responses to a series of hypothetical treatment-choice questions, it is possible to infer the tradeoffs patients are willing to make among treatment attributes.

**Table 1:** Attributes and levels in the treatment-choice questions

Attribute	Attribute label	Levels
Probability of optimal laboratory values	Chance that the treatment keeps your labs within their recommended ranges	▪ 80 out of 100 (80%) ▪ 60 out of 100 (60%) ▪ 25 out of 100 (25%)
Probability of symptom relief	Chance that the treatment relieves your SHPT symptoms	▪ 75 out of 100 (75%) ▪ 35 out of 100 (35%) ▪ 5 out of 100 (5%)
Risk of hypocalcemia	Risk of low blood calcium	▪ 0 out of 100 (0%) ▪ 2 out of 100 (2%) ▪ 10 out of 100 (10%)
Severity of nausea and vomiting	Nausea and vomiting (medication alternatives only)	▪ None ▪ Mild ▪ Moderate
Mode of administration	How you receive the medicine (medication alternatives only)	▪ Given through dialysis line during your regular dialysis treatment ▪ Pill once a week ▪ Pill once a day
Cost	Out-of-pocket cost of treatment	▪ $50 per month (medication alternatives only) ▪ $100 per month (medication alternatives only) ▪ $200 per month (medication alternatives only) ▪ $500 one time (surgery only)

*SHPT* secondary hyperparathyroidism

### Survey instrument development

Five steps were involved in developing the DCE survey instrument: a literature review, a review of patient resources issued by the National Kidney Foundation (NKF), nephrologist input, and a focus group and qualitative pretest interviews with ESRD patients with SHPT to ensure that the survey questions were clear and comprehensible and to identify any refinements that were necessary before administration of the survey. The first three steps informed the preliminary selection of potentially relevant SHPT attributes. The Additional file 1: appendix provides additional details about the focus group and qualitative pretests and presents the final survey instrument.

**Fig. 1:**
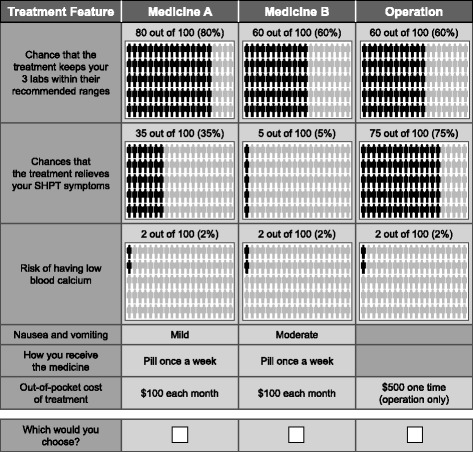
Example treatment-choice question. In the survey instrument, each choice question included a combination of the attribute levels presented in Table 1

The DCE survey instrument, consistent with good research practices [9], was developed to elicit respondents’ preferences for three hypothetical treatment options—surgery and two medication alternatives—in a series of questions. The hypothetical treatments were defined by efficacy, safety and tolerability, mode of administration, and out-of-pocket cost attributes (Table 1). The attributes and levels were chosen to represent the features of SHPT management that are relevant and salient to respondents and that differentiate existing disease-management options. Figure 1 presents an example of a treatment-choice question.

**Fig. 2:**
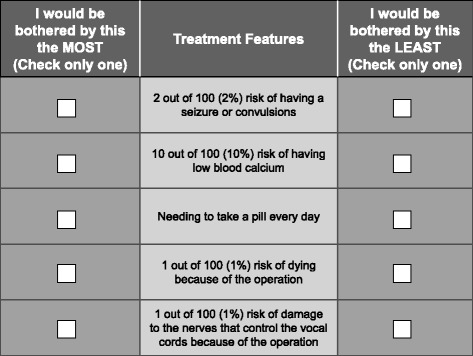
Example of a best-worst scaling question

The survey instrument included additional questions to evaluate respondents’ preferences for treatment features that were not fully explored in the DCE. For example, surgery has a number of characteristics that were described to respondents but were not included as separate attributes in the DCE because these characteristics are specific to surgery and do not vary among medication alternatives (e.g., incision, hospitalization, anesthesia). An object-case best-worst scaling (BWS) exercise was included in the survey instrument to quantify the relative bother of these characteristics. The items in the BWS exercise included nine attributes associated with surgery and one attribute associated with medication, which provided a link between the DCE and BWS results (Table 2). In each BWS question, respondents were presented with a list of five treatment attributes and asked to state which attribute would be most and least bothersome. Figure 2 presents an example BWS question. In addition, because the available SHPT medications differ primarily by mode of administration, the survey instrument included a question about whether respondents would prefer to receive an SHPT medication orally once per day, orally once per week, or through a dialysis line. Patients who preferred administration through a dialysis line, a novel form of administration in this indication, were asked to explain why; these free-text responses were analyzed qualitatively [10]. The survey instrument also included demographic questions and disease history and treatment questions.

**Table 2:** Attributes included in the best-worst scaling questions

Attributes	
Having a cut from 1 to 2 in. long on your neck	
Needing to stay in the hospital overnight after the operation	
Being under general anesthesia during the operation	
1 out of 300 (0.3%) risk of serious bleeding during the operation	
1 out of 100 (1%) risk of damage to the nerves that control the vocal cords because of the operation	
5 out of 100 (5%) risk of having a hoarse voice for up to six months after the operation	
1 out of 100 (1%) risk of dying because of the operation	
2 out of 100 (2%) risk of having a seizure or convulsions	
10 out of 100 (10%) risk of having low blood calcium	
Needing to take a pill every day

To prepare the survey for online administration, the experimental design was developed following good research practices [11]. The SAS implementation of a commonly used D-optimal algorithm was used to construct a fractional factorial experimental design for the medication alternatives and surgery in each choice question [12, 13]. The final experimental design included 48 DCE questions divided into 6 blocks, each with 8 questions. Each respondent was randomly assigned to answer the choice questions in one block. The pattern of responses to such a series of questions provided information that was used to estimate the extent to which changes in the levels of treatment attributes affected treatment choice.

### Study population

Individuals in the United States registered as patients in NKF’s member database were invited to be screened for study eligibility through an e-mail invitation that explained the study. To be eligible, respondents were required to be aged 18 years or older, to have self-reported ESRD, to be undergoing in-center hemodialysis, and to have not undergone a parathyroidectomy. Respondents were not required to have SHPT to complete the survey. All study participants provided informed consent. Participants who completed the survey were provided with a $25 gift card as compensation for their time and effort. The study was approved by the Office of Research Protection and Ethics at RTI International and complied with the Declaration of Helsinki.

### Statistical analyses

#### Discrete-choice experiment analyses

The DCE data were analyzed using a random-parameters logit (RPL) model following good research practices [14]. This model yielded a relative preference parameter for each attribute level (Table 1). The parameter estimates from RPL models can be interpreted as preference weights indicating the relative strength of preference for each attribute level. An alternative-specific constant was included in the model to estimate a preference parameter for the surgery alternative. Out-of-pocket cost of the medicine was modeled as an interaction between the cost level shown for each alternative and the natural log of the respondent’s reported household income in the previous calendar year. The other treatment attributes were modeled as categorical, effects-coded variables [14, 15]. To identify potential interaction effects between surgery and the attributes that varied across both the medication alternatives and surgery (i.e., probability of optimal laboratory values, probability of symptom relief, risk of hypocalcemia), we also interacted each level of these attributes with the alternative-specific constant to capture potential differences in preferences for outcomes depending on whether they were achieved medically or surgically. This interaction was conducted because the effects of surgery are essentially permanent and the effects of medication continue only as long as the medication is administered. The hypothesis was, therefore, that the positive aspects of surgery would be better (i.e., more important) than the positive aspects of medication, and the negative aspects of surgery would be worse than the negative aspects of medication. To determine the conditional relative importance of an attribute, the difference between the attribute level with the highest preference weight and the level with the lowest preference weight was calculated. Finally, the estimated preference weights were used to estimate the preference shares, or the probability that patients would prefer each of the three available types of SHPT treatment: surgical, oral, and intravenous.

#### Best-worst scaling analyses

The analysis of the BWS data assumed that choices recorded from BWS questions reflected two independent decisions. Importance weights were estimated using an RPL model that related respondents’ choices for the most and least bothersome items to the item-specific variables. The items were effects coded. RPL controls for the correlation of multiple responses from the same respondent that are introduced by requiring the respondent to make two choices (i.e., the most bothersome item and least bothersome item) in each BWS question. Importance weights represent the relative weights respondents placed on each item when selecting the most bothersome item. Larger coefficients indicate that the item was more bothersome. Conversely, smaller coefficients indicate that the item was less bothersome. To calculate estimates of relative bother from the importance weights, we used a probability-based rescaling procedure [16].

#### Qualitative analyses

Among patients who preferred administration of an SHPT medication through a dialysis line, we conducted thematic analysis of the reasons provided by respondents and calculated the frequency with which certain concepts were reported [10].

## Results

### Respondent characteristics

The target sample was 200 respondents. A total of 7582 individuals were invited by e-mail to be screened for eligibility, and 500 individuals accessed the survey. Of those who accessed the survey, 231 (3% of those who were invited and 46% of those who accessed the survey) were eligible. Of those who were eligible, 219 (95%) consented to participate. Of those who consented to participate, 200 (91%) completed the survey. Table 3 summarizes respondents’ demographic and clinical characteristics.

**Table 3:** Respondent characteristics

Characteristic	Statistic or category	Overall (*N* = 200)
*Demographic characteristics*		
Age	Mean (SD)	54.2 (14.0)
	Missing	2
What is your gender?	Female	102 (51.0%)
	Male	96 (48.0%)
	Prefer not to answer	2 (1.0%)
How would you describe your race or ethnicity?	White or Caucasian	120 (60.0%)
	Black or African American	52 (26.0%)
	Other	27 (13.5%)
	Prefer not to answer	6 (3.0%)
What is your marital status?	Married / living as married / civil partnership	89 (44.5%)
	Not married	109 (54.5%)
	Prefer not to answer	2 (1.0%)
What is the highest level of education you have completed?	High school or equivalent (e.g., GED) or less	40 (20.0%)
	More than high school	156 (78.0%)
	Prefer not to answer	4 (2.0%)
Which of the following best describes your employment status?	Employed full-time	24 (12.0%)
	Retired	53 (26.5%)
	Disabled / unable to work	96 (48.0%)
	Other	26 (13.0%)
	Prefer not to answer	1 (0.5%)
What was your total household income before tax and other deductions in 2014?	Less than $20,000	57 (28.5%)
	$20,000 to $29,999	41 (20.5%)
	More than $29,999	75 (37.5%)
	Don’t know / not sure	2 (1.0%)
	Prefer not to answer	25 (12.5%)
*Clinical characteristics*		
How long have you been receiving dialysis?	Less than 6 months	2 (1.0%)
	6 months to less than 1 year	26 (13.0%)
	1 year to less than 2 years	41 (20.5%)
	2 years to less than 5 years	78 (39.0%)
	5 years to less than 10 years	37 (18.5%)
	10 years or more	16 (8.0%)
Have you previously received a kidney transplant?	Yes	20 (10.0%)
	No	180 (90.0%)
Are you on a kidney transplant waiting list?	I am currently on a kidney transplant waiting list	63 (31.5%)
	I am in the process of getting on a kidney transplant waiting list	53 (26.5%)
	I am not on a kidney transplant waiting list	84 (42.0%)
Which of the following problems have you ever experienced because of your kidney disease?	Anemia (low hemoglobin)	155 (77.5%)
	Bleeding in the stomach or intestines	9 (4.5%)
	Bone, joint, or muscle pain	97 (48.5%)
	Muscle weakness	115 (57.5%)
	Weakening of bones or bone fractures	19 (9.5%)
	Changes in blood sugar (glucose)	50 (25.0%)
	Fluid buildup in the lungs	54 (27.0%)
	Hepatitis B, hepatitis C, or liver failure	3 (1.5%)
	High blood pressure, heart attack, or heart failure	112 (56.0%)
	High potassium levels	91 (45.5%)
	Lack of appetite or poor nutrition	76 (38.0%)
	Nerve damage or nervous system problems (such as restless legs syndrome)	68 (34.0%)
	Seizures	9 (4.5%)
	Skin infection	20 (10.0%)
	Stroke	5 (2.5%)
	Swelling or edema	97 (48.5%)
	None of the above	5 (2.5%)
Has a doctor or other health care professional ever told you that you have secondary hyperparathyroidism (SHPT)?^a^	Yes	49 (24.5%)
	No	104 (52.0%)
	Don’t know / not sure	47 (23.5%)
If all SHPT medicines worked equally well, how would you choose to receive the medicine?	Pill once a day	30 (15.0%)
	Pill once a week	19 (9.5%)
	Given through dialysis line during your regular dialysis treatment	151 (75.5%)

Percentages do not include missing responses in the denominator

*PTH* parathyroid hormone, *SD* standard deviation, *SHPT* secondary hyperparathyroidism

^a^Respondents were not required to have SHPT to complete the survey

### Preference weights and conditional relative importance of treatment attributes

**Fig. 3:**
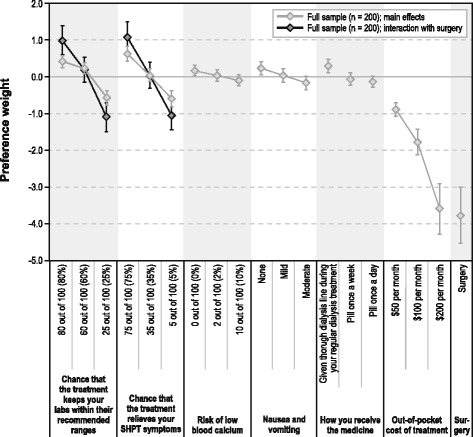
Secondary hyperparathyroidism treatment preference weights. SHPT = secondary hyperparathyroidism. The vertical bars surrounding each mean preference weight denote the 95% confidence interval about the point estimate. If the confidence intervals do not overlap for pairs of levels in a particular attribute, the parameter estimates are statistically different from each other at the 5% level of significance

Figure 3 presents the preference weights. More preferred outcomes have higher preference weights. Regardless of whether the probability of optimal laboratory values was interacted with surgery, an 80% chance was preferred to a 60% chance for this attribute, and a 60% chance was preferred to a 25% chance. Similarly, no or less-severe nausea and vomiting was preferred to more-severe nausea and vomiting. The vertical distance between preference weights within an attribute represents the relative importance of moving from one level to another. For example, the change from *pill once a week* to *given through dialysis line during your regular dialysis treatment* had a relative importance of 0.36 (= 0.267 − [−0.095]) (95% confidence interval [CI], 0.09–0.64). The change from *pill once a day* to *pill once a week* had a relative importance of 0.08 (= −0.095 − [−0.172]) (95% CI, −0.16-0.31), and the change from *pill once a day* to *given through dialysis line during your regular dialysis treatment* had a relative importance of 0.44 (= 0.267 − [−0.172]) (95% CI, 0.18–0.70).

Comparing across attributes, the change in the probability of symptom relief (as a result of medication) from 35% to 75% had a relative importance of 0.58 (= 0.597–0.022) (95% CI, 0.31–0.84), whereas the change from *pill once a day* to *given through dialysis line during your regular dialysis treatment* had a relative importance of 0.44 (as mentioned previously). Therefore, the change in the probability of symptom relief (as a result of medication) from 35% to 75% was approximately 1.3 (= 0.58 ÷ 0.44) times as important as the change from *pill once a day* to *given through dialysis line during your regular dialysis treatment*. When efficacy outcomes were achieved as a result of surgery, preference weights reflected stronger preferences.

The difference in the preference weights of the most and least preferred level of an attribute is a measure of the conditional relative importance of the attribute over the range of levels included. Avoiding surgery had the greatest conditional relative importance (3.79 [95% CI, 3.05–4.52]), followed by out-of-pocket cost of treatment (2.71 [95% CI, 2.21–3.21]). The efficacy outcomes resulting from surgery were relatively more important to respondents than the same outcomes resulting from medication. The probability of symptom relief (as a result of surgery) had a conditional relative importance of 2.13 (95% CI, 1.44–2.81), and the probability of symptom relief (as a result of medication) had a conditional relative importance of 1.22 (95% CI, 0.86–1.58). The probability of optimal laboratory values (as a result of surgery) had a conditional relative importance of 2.08 (95% CI, 1.38–2.78), and the probability of optimal laboratory values (as a result of medication) had a conditional relative importance of 1.00 (95% CI, 0.68–1.33). The remaining three attributes—mode of administration, severity of nausea and vomiting, and risk of hypocalcemia—were statistically significantly relatively less important than the other attributes given the ranges of the attribute levels included in the study. In addition, preferences for avoiding hypocalcemia did not differ between the medication and surgical alternatives.

### Preference shares

Preference weights also can be used to estimate preference shares for treatment profiles defined by a given combination of attribute levels. The preference share for each treatment profile represents the estimated probability that the treatment would be chosen from among a set of treatments. Preference shares were calculated for two scenarios (Table 4). Given the choice between a medicine administered as a daily pill and a medicine administered through the dialysis line, there was a 61% probability that a patient would choose to receive the medicine through the dialysis line, all else equal. Likewise, given the choice among medicine given as a daily pill, a medicine given through the dialysis line, and surgery, there was a 60% probability that a patient would choose to receive medication through the dialysis line, all else equal.

**Table 4:** Preference shares

	Surgery or Medication	Chance That the Treatment Keeps Your Labs Within Their Recommended Ranges	Chance that the Treatment Relieves Your HPT Symptoms	Risk of Low Blood Calcium	Nausea and Vomiting	How You Receive the Medicine	Out-of-Pocket Cost of Treatment per Month	Predicted Preference Share (95% CI)
Scenario 1: two medications, $15 out-of-pocket cost								
Treatment profile 1	Medication	60 out of 100 (60%)	35 out of 100 (35%)	2 out of 100 (2%)	Mild	Given through dialysis line during your regular dialysis treatment	$15	60.8% (54.6%–67.0%)
Treatment profile 2	Medication	60 out of 100 (60%)	35 out of 100 (35%)	2 out of 100 (2%)	Mild	Pill once a day	$15	39.2% (33.0%–45.4%)
Scenario 2: two medications and surgery, $15 out-of-pocket cost of medications								
Treatment profile 1	Medication	60 out of 100 (60%)	35 out of 100 (35%)	2 out of 100 (2%)	Mild	Given through dialysis line during your regular dialysis treatment	$15	60.0% (53.8%–66.2%)
Treatment profile 2	Medication	60 out of 100 (60%)	35 out of 100 (35%)	2 out of 100 (2%)	Mild	Pill once a day	$15	38.7% (32.6%–44.8%)
Treatment profile 3	Surgery	60 out of 100 (60%)	35 out of 100 (35%)	2 out of 100 (2%)	[Not applicable]	[Not applicable]	$0 (surgery is defined as a one-time cost of $500, which is reflected in the preference weight for surgery)	1.3% (0.1%–2.5%)

*CI* confidence interval, *SHPT* secondary hyperparathyroidism

### Best-worst scaling

**Fig. 4:**
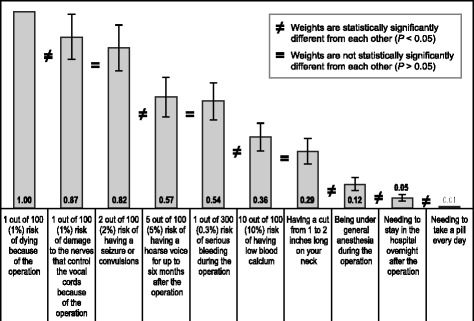
Best-worst scaling relative bother estimates (*N* = 200). The = symbol between two weights indicates that those two weights are not statistically significantly different from each other (*P* < 0.05), and the ≠ symbol indicates that those two weights are statistically significantly different from each other. For example, *1 out of 100 (1%) risk of dying because of the operation* is statistically significantly different from *1 out of 100 (1%) risk of damage to the nerves that control the vocal cords because of the operation*, whereas *1 out of 100 (1%) risk of damage to the nerves that control the vocal cords because of the operation* is not statistically significantly different from *2 out of 100 (2%) risk of having a seizure or convulsions*. The bars surrounding each mean importance weight denote the 95% confidence interval about the point estimate

Figure 4 summarizes the estimated bother of the treatment attributes included in the BWS questions. The most bothersome treatment attribute was a 1% surgical mortality risk. The least bothersome attribute was daily oral administration.

### Qualitative responses

Among respondents who preferred to receive an SHPT treatment through a dialysis line rather than orally (*n* = 151; 75.5%), the major themes that emerged centered on improved convenience (*n* = 59; 39.1%) (e.g., “I wouldn’t need to remember to take pill”; “Most convenient since I am already there [at the dialysis facility]”), greater provider involvement (*n* = 25; 16.6%) (e.g., “It would be controlled by the nurses”; “They monitor and adjust the medicine accordingly”; “So I have a treatment team there to monitor me”), and issues with pills (*n* = 18; 11.9%) (e.g., “one less pill of 11 I already take”; “Really don’t like taking pills”).

## Discussion

Patients with ESRD have discernible preferences regarding treatment options for SHPT. Because patients distinguished between outcomes resulting from surgery and the same outcomes resulting from medication, our results suggest that patients understand the clinical implications of treating SHPT medically rather than surgically and that, on average, patients prefer medical management of SHPT to surgery. The most bothersome attribute of surgery was the risk of surgical mortality. In addition, our findings suggest that patients would prefer receiving SHPT medication intravenously through the dialysis line compared with receiving daily or weekly pills, commonly for reasons relating to convenience and increased provider involvement in the medication-delivery process.

ESRD and SHPT are clinically complex disorders that entail difficult therapeutic decision making. Moreover, there is a prevailing notion in ESRD care that low health literacy may limit patients’ ability to be involved in choice of therapies [17]. Although it can be difficult to distinguish SHPT symptoms from the other symptoms of ESRD and the effects of dialysis, we found that patients with SHPT who participated in the focus group and pretest interviews understood the implications of laboratory values and the consequences of SHPT. In addition, the results of the survey indicate that respondents, whether or not they had SHPT, understood the information about SHPT included in the survey and demonstrated a willingness to trade off among the medications and surgery and the attributes of each. Overall, we were able to engage patients in identifying their treatment preferences, and participants in this study were well informed about treatment attributes and about the management of SHPT.

In clinical practice, physicians and nurses treating patients with ESRD encounter time constraints and potential barriers to shared decision making. Our study provides a first glimpse at what providers may learn if they were to elicit preferences for SHPT treatment from their patients. Providers wishing to engage patients in shared decision making could use the attributes we identified as a reasonable starting point for discussions of which SHPT treatment may best meet a patient’s needs and values. Beyond our study, a variety of resources are available to empower patients in articulating their treatment preferences and to support providers as they partner with patients in making treatment decisions (e.g., patient-education materials issued by the NKF and strategies for providers to optimize patients’ health literacy, such as teach-back techniques and the Ask Me 3® patient-education program [18–20]). Further, the Renal Physicians Association and the American Society of Nephrology jointly issued a clinical practice guideline on shared decision making in the initiation of and withdrawal from dialysis [21]. Although providers and patient advocacy groups are making strides in this area, more work will be required to fully engage patients with ESRD in shared decision making [2].

A number of limitations must be considered when the results of this study are interpreted. Respondents were asked to evaluate hypothetical treatments, and not all potential attributes of SHPT treatments were included. Thus, the results relate only to those attributes included in the survey. Nevertheless, the attributes were informed by the scientific literature and focus group discussions with patients, to help ensure that the most relevant SHPT attributes were evaluated. In addition, the results are subject to selection bias, given that study respondents represented a convenience sample recruited from an opt-in database of NKF members. Responses to the e-mail invitation to participate in the study assumed computer access and literacy, and the engagement and health literacy of the largely well-educated sample may not reflect those of the overall ESRD population. Thus, representativeness of the population of individuals undergoing hemodialysis and experiencing SHPT cannot be ensured, and the characteristics of patients who were invited to participate but chose not to were not analyzed. However, the study respondents’ major demographic characteristics—including age, gender, and race/ethnicity—are generally comparable with those of US patients undergoing dialysis [22]. Finally, the study data represent average preferences among participants in this study. Patients’ specific preferences will vary, and the same analyses conducted with a different sample could yield different findings.

## Conclusions

We found that patients with ESRD who are undergoing hemodialysis understand SHPT and have clear and measureable treatment preferences. Providers focused on patient-centered care and patient satisfaction may find our survey and results useful as a starting point in daily clinical practice as they work to align their care decisions with their patients’ preferences.

## Additional file

Additional file 1: Appendix. Development of the Survey Instrument section: Describes the focus group and pretest interviews that informed survey development. Focus Group Discussion Guide section: Presents the discussion guide used during the focus group. Secondary Hyperparathyroidism Treatment Preference Survey section: Presents the survey instrument. (DOCX 306 kb)

## Abbreviations

BWS: best-worst scaling
CI: confidence interval
DCE: discrete-choice experiment
ESRD: end-stage renal disease
NKF: National Kidney Foundation
RPL: random-parameters logit
SHPT: secondary hyperparathyroidism
US: United States.
