# Supplementary material for: Hemodialysis patients’ preferences for the management of secondary hyperparathyroidism
Source: BMC Nephrol. 2017 Jul 28;18:254. doi: 10.1186/s12882-017-0665-8 (PMC5532768; doi:10.1186/s12882-017-0665-8)
Supplement: Additional file 1: — Appendix. Development of the Survey Instrument section: Describes the focus group and pretest interviews that informed survey development. Focus Group Discussion Guide section: Presents the discussion guide used during the focus group. Secondary Hyperparathyroidism Treatment Preference Survey section: Presents the survey instrument. (DOCX 306 kb) [file 12882_2017_665_MOESM1_ESM.docx]

**Supplementary Appendix**

# DEVELOPMENT OF THE SURVEY INSTRUMENT

## Focus Group

To assess patients’ understanding and perceptions of secondary hyperparathyroidism (SHPT) and its treatments and to further guide selection of attributes for the survey instrument, a focus group was conducted with a convenience sample of 6 patients with SHPT undergoing hemodialysis, who were recruited by a qualitative research facility in Raleigh, North Carolina. Individuals were eligible to participate in the focus group if they were 18 years of age or older, currently undergoing dialysis, and had a self-reported physician diagnosis of SHPT.

Participants reported in writing the three most favorable and three least favorable attributes of their SHPT treatment and the one attribute they would change about their current treatment. Participants’ (deidentified) responses were displayed for a group discussion. Participants then took part in a ranking exercise to identify the five best and five worst attributes from a set of attributes identified by the participants or hypothesized by the authors to be of interest. The focus group discussion and ranking exercise confirmed that no important treatment attributes were missing from the preliminary list and that patients understood the attributes.

## Pretest Interviews

Before online administration, the survey instrument underwent qualitative pretesting in face-to-face interviews with a convenience sample of 10 patients with end-stage renal disease (ESRD) and SHPT undergoing hemodialysis, who were recruited by a qualitative research facility in Raleigh, North Carolina. Interview participants were asked to think aloud as they completed the draft survey. Participants were also asked a series of debriefing questions to determine whether they understood the definitions and instructions, accepted the hypothetical context of the survey, and successfully completed the choice questions as instructed. The survey instrument was refined based on the results of the pretest interviews.

# FOCUS GROUP DISCUSSION GUIDE

Introduction (~10 minutes)

**[Introduce moderator and note-taker. Review consent form, which will explain the purpose and format of the interview.]**

We are working with a biotechnology company to learn more about patient experiences with treatments for moderate to severe secondary hyperparathyroidism (SHPT). Moderate to severe SHPT means the parathyroid glands are making too much parathyroid hormone. Treatments for SHPT include oral medicines and surgery.

Today we will ask you some questions about

- Your ***experiences*** with SHPT treatments,
- What things you ***like and dislike*** about these treatments, and
- What you ***would change*** about these treatments if you could.

Please keep in mind that there are no wrong answers, so please share your opinions freely. You are the experts today, and we want to learn from you and your experiences.

During the focus group, we ask that you use only your first name, and please do not share any personal information such as your age, your doctor’s name, or where you live. We will not be recording any of this information in our notes. Your privacy is important to us, and we want to be extra careful that no one shares or feels obligated to share personal information unnecessarily.

**[Ask participants to read the informed consent form. Answer any questions regarding the informed consent form. Ask participants to sign the informed consent form. Then collect the signed informed consent forms and make sure that each participant has a copy of the informed consent form to keep.]**

**[Assign a participant number to each participant based on his or her position around the table starting with the first participant sitting to the left of the moderator and moving clockwise around the table. This participant number is for the research team’s use to be able to match the results from the index card feedback to the corresponding participant and will not be disclosed to the participant.]**

Treatment History (~10 Minutes)

First, we’d like to learn a little more about your experiences with SHPT and its treatment.

To begin with, will each of you tell me and the group

- When were you first diagnosed with SHPT?
- What types of treatments you have taken since you were first diagnosed?
- How many of you have taken only one type of treatment since you were diagnosed? How many of you have taken more than one type of treatment? Can you tell me a little bit about the differences you noticed with the different types of treatments? **[Facilitator to probe regarding first and second lines of treatment and when new treatments were initiated versus participants experiencing only one type of treatment at various times.]**

We do not need to know the names of specific treatments. We are more interested in the types of treatments you have taken.

**[If no participants have mentioned surgery, ask participants if anyone has had surgery to treat SHPT.]**

**[Ask all participants if their doctor has spoken to them about surgery as a possible treatment for SHPT.]**

Treatment Likes and Dislikes (~20 minutes)

Today, we want to learn more about your experiences and factors that contribute to your satisfaction with SHPT treatments.

**[Hand out three cards to each participant. Each card should have the participant number listed at the top of the card.]**

Each of you has three cards.

On one card, please list the three things you ***like most*** about your current treatment. Please try to list three things; however, if you cannot think of three things, that is okay.

**[Collect the card containing the likes from each participant.]**

On the second card, please list the three things you ***dislike most*** about your current treatment. Please try to list three things; however, if you cannot think of three things, that is okay.

**[Collect the card containing the dislikes from each participant.]**

**[Write the likes on the flip chart (duplicates from multiple participants should not be written twice; however, the number of participants providing the same response can be indicated next to the response). When writing the items on the flip chart, ask for clarification if the response is not specific enough. For example, if the card states, “treatment works well,” ask the group what “works well” means.]**

**[Write the dislikes on the flip chart (duplicates from multiple participants should not be written twice; however, the number of participants providing the same response can be indicated next to the response). Again, please ask for clarification or specification if items are general or vague.]**

**[Ask participants if there are any features not listed on the flip chart that they feel should be listed.]**

Unmet Needs (~10 minutes)

On the third card, please write down your response to the following question:

- If there was one thing you **could change** about your current treatment, what would it be?

**[After everyone has completed his or her card…]**

We would now like you to share with the group the thing you would like to change about your current treatment.**[Ask each participant to tell the group their answer. Write down answers on a flip chart (duplicates from multiple participants should not be written twice; however, the number of participants providing the same response can be indicated next to the response). Again, please ask for clarification or specification if items are general or vague.]**

Discussion of Treatment Attribute Concepts

[This section will only be used if participants are unable to identify SHPT treatment experiences, for instance if they are unaware of SHPT-specific treatments separate from other care/treatment that they may be receiving. If so, this section will provide an opportunity to discuss hypothesized treatment concepts that will then be incorporated into the BWS exercise.]

Let’s discuss some potential benefits as well as drawbacks for a new medication. [Probe on the below concepts]

- Efficacy of the treatment (probe on value of improved “lab values”: lower levels of PTH (parathyroid hormone), P (phosphorus) and CA (calcium) as well as CA X P. Have they heard of these before? What if lab values improve but no other effects?
- Downstream cardiovascular or other risks if SHPT not controlled.
- Risks associated with surgery (e.g. complications).
- Risk of hypocalcaemia (caused by loss of calcium from or insufficient entry of calcium into the circulation). Have patients heard of this term or when described as above, recall discussion of this risk with their physician?
- Nausea and vomiting (reduction in the number of episodes per week)
- Treatment formulation
- Oral daily pill taken at home
- Medicine injected into access port during hemodialysis by nurse
- One-time surgery (parathyroidectomy)

Break (~10 minutes)

**[During the break, enter 12 to 20 of the best and worst items into the best-worst scaling spreadsheet. Items in the best-worst scaling spreadsheet will include items raised by the focus group participants in the previous exercises in addition to items identified by the project team prior to the focus groups.]**

Best-Worst Scaling Exercise (~20 minutes)

Thank you all for sharing your opinions about your current SHPT treatment experience and what you think could be better. We have one more exercise we would like you to help us with.

**[Hand out the best-worst scaling worksheet to each participant. Each worksheet should include the participant’s identification number.]**

We will now show you a few slides. On each slide, you will see five features of SHPT treatments. Each feature has a number associated with it.

**[Show the first best-worst scaling slide.]**

In front of you there is a piece of paper with slide numbers listed down the left side and two columns for each slide number. One column is labeled “Best” and one column is labeled “Worst.”

For each slide, please think about which of the five features is the best one and which of the five features is the worst one.

Keep in mind that sometimes all five features could be considered bad. In that case, the best feature is the one that you think is the least bad. Likewise, there could be slides on which all features could be considered good. In that case, the worst feature you think is the one that is the least good.

Now, please look at the paper in front of you. Once you have identified the feature on the slide that you think is best, please put the number corresponding to that feature in the “Best” column on the line corresponding to that slide number. Likewise, once you have identified the feature on the slide that you think is worst, please put the number corresponding to that feature in the “Worst” column on the line corresponding to that slide number.

Does anyone have any questions about the task we have asked you to do?

**[Answer participant questions to ensure that participants understand the task.]**

There are 11 more slides like this. For each slide, let’s do the same thing you did for the first slide. Let’s begin.

**[Present each slide and ask participants to record the best and worst for each slide until all slides have been presented.]**

Thank you. Please pass your papers back to me.

Summing Up (~10 minutes)

Thank you for sharing your thoughts and experiences with us today. Is there anything related to your current SHPT treatment that you would like to share with us?

**[Discuss issues raised.]**

Your input has been very helpful to us. Thank you again!

# SECONDARY HYPERPARATHYROIDISM TREATMENT PREFERENCE SURVEY

Dialysis Survey

Thank you for agreeing to take this survey about dialysis. First, we would like to ask you a few questions about your experience with dialysis.

1. How long have you been receiving dialysis?

- Less than 6 months
- 6 months to less than 1 year
- 1 year to less than 2 years
- 2 years to less than 5 years
- 5 years to less than 10 years
- 10 years or more

1. Have you previously received a kidney transplant?

- Yes
- No

1. Are you on a kidney transplant waiting list?

- I am currently on a kidney transplant waiting list
- I am in the process of getting on a kidney transplant waiting list
- I am not on a kidney transplant waiting list

1. Which of the following problems have you ever experienced because of your kidney disease? *(Check all that apply)*

- Anemia (low hemoglobin)
- Bleeding in the stomach or intestines
- Bone, joint, or muscle pain
- Muscle weakness
- Weakening of bones or bone fractures
- Changes in blood sugar (glucose)
- Fluid buildup in the lungs
- Hepatitis B, hepatitis C, or liver failure
- High blood pressure, heart attack, or heart failure
- High potassium levels
- Lack of appetite or poor nutrition
- Nerve damage or nervous system problems (such as restless legs syndrome)
- Seizures
- Skin infection
- Stroke
- Swelling or edema
- None of the above

Secondary Hyperparathyroidism

People who are on dialysis may develop a condition known as secondary hyperparathyroidism (SHPT).

When your kidneys fail, your parathyroid glands (4 small glands in your neck) may make too much parathyroid hormone (PTH). Too much PTH can cause high levels of calcium and phosphorus to be released into the bloodstream.

Some patients with SHPT may not feel any symptoms, but other patients might feel symptoms like:

- Dry or itchy skin
- Pain in muscles, joints, and/or bones
- Stiff joints
- Muscle weakness

It is important to keep the levels of PTH, calcium, and phosphorus in your bloodstream within their recommended ranges. It can be harmful to your health if 1 of these levels is too high or too low, even if the other 2 levels are in the recommended ranges. If your levels of calcium and phosphorus are too high, you may have an increased risk of “bone and mineral disease” that can cause bone pain and weakness.

The goal of SHPT treatment is to keep the amount of PTH, calcium, and phosphorus in your bloodstream within the recommended ranges. Keeping all 3 of these levels within their recommended ranges helps reduce your risk of getting bone and mineral disease.

Your Experience With SHPT

1. Has a doctor or other health care professional ever told you that you have secondary hyperparathyroidism (SHPT)?

- Yes
- No
- Don’t know / not sure

1. Which of the following symptoms of SHPT have you experienced?

- Dry or itchy skin
- Pain in muscles, joints, and/or bones
- Stiff joints
- Muscle weakness
- None of the above

1. Have you taken cinacalcet (Sensipar^®^) within the past 2 years to treat SHPT?
   - Yes
   - No
   - Don’t know / not sure

Parathyroidectomy (operation to remove some or all of your parathyroid glands)

SHPT can be treated in 2 ways: taking medicines or having an operation.

An operation to treat SHPT is called a *parathyroidectomy*. If you have this type of operation, a doctor will remove some or all of the 4 parathyroid glands in your neck. The doctor will make a cut from 1 to 2 inches long on the middle to lower part of your neck in a spot matching the skin folds on your neck. The doctor will then remove 1 or more of the parathyroid glands and close the cut on your neck with stitches. The operation is performed under general anesthesia in the hospital. You will need to stay in the hospital overnight after the operation before you can go home.

This type of operation will reduce levels of PTH in your bloodstream. However, there are risks associated with the operation:

- There is a 1 out of 300 (0.3%) risk of serious bleeding with the operation. In some cases, this type of bleeding may mean that you need a blood transfusion.
- There is a 5 out of 100 (5%) risk that your voice will be hoarse (harsh, raspy, or strained) for up to 6 months after the operation. There is a 1 out of 100 (1%) chance that your voice will be hoarse permanently because of damage to the nerves that control your vocal cords.
- Approximately 1 out of 100 people (1%) who have this operation will die because of the operation.

Please indicate whether each statement below is true or false.

|  | | | True | False | | |
| --- | --- | --- | --- | --- | --- | --- |
| 1. During a parathyroidectomy, the doctor removes 1 or more of the parathyroid glands. |  | | |  |  |  |
| 1. There is no chance of permanent hoarseness because of the operation. |  | | |  |  |  |

Features of SHPT Treatments

In the next few pages, we will describe features of different SHPT treatments (both an operation and medicines). This information will help you answer questions later in the survey. You can refer back to this information as you take the survey.

Even if you have never been told by a doctor that you have SHPT, we are still interested in your opinions about features of SHPT treatments.

Treatment Feature: Chance That the Treatment Keeps All Three Labs Within their Recommended Ranges

SHPT treatments (an operation or medicines) can help lower the levels of PTH, calcium, and phosphorus in your bloodstream. An SHPT treatment works if it lowers the levels of PTH, calcium, and phosphorus in your bloodstream to the recommended ranges. The levels of PTH, calcium, and phosphorus in your bloodstream are measured using a blood test. The results of the blood test are called *lab values*. It is important to keep all 3 lab values in their recommended ranges at the same time.

If you have an operation to remove 1 or more of your parathyroid glands from your neck and your lab values are lowered to the recommended ranges, then you will not need additional treatment.

If you take an SHPT medicine and your lab values are lowered to the recommended ranges, you will need to continue taking the medicine to keep your lab values in the recommended ranges.

1. Which of the following is most important to you? *(Check only 1)*

- Keeping your PTH lab value within the recommended range
- Keeping your calcium lab value within the recommended range
- Keeping your phosphorus lab value within the recommended range
- Keeping levels for all 3 lab values (PTH, calcium, and phosphorus) within their recommended ranges

Helping You Think About the Chance That the Treatment Keeps All Three Lab Values Within Their Recommended Ranges

We will use pictures to help you think about how many patients will have their lab values in the recommended ranges because of an SHPT treatment (an operation or a medicine).

Each figure in the picture below represents 1 person who has an operation or takes a medicine to treat SHPT. There are 100 figures in the picture. The picture shows the chance that the SHPT treatment keeps all 3 lab values within their recommended ranges in 100 people. The treatment **would** keep all 3 lab values within their recommended ranges for the figures shown in color. The treatment **would not** keep all 3 lab values within their recommended ranges for the figures in gray.

In this example:

- The figures in color show that the treatment **would** keep all 3 lab values within their recommended ranges for 75 people out of 100 (75%).
- The gray figures show that the treatment **would not** keep all 3 lab values within their recommended ranges for 25 people out of 100 (25%).

When there are more figures in color, your chance of keeping all 3 lab values within their recommended ranges is higher.

**Example 1:**


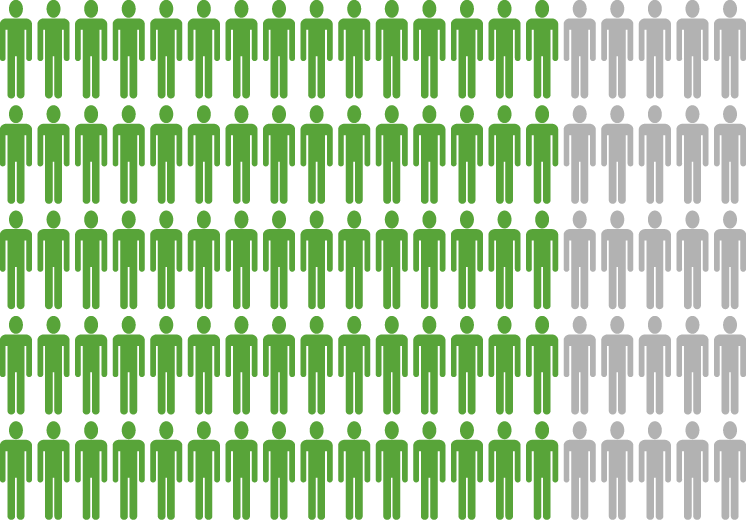


**There are 75 figures in color. That means that the treatment would keep all 3 lab values within their recommended ranges for 75 people out of 100 (75%).**

Please look at the picture below:


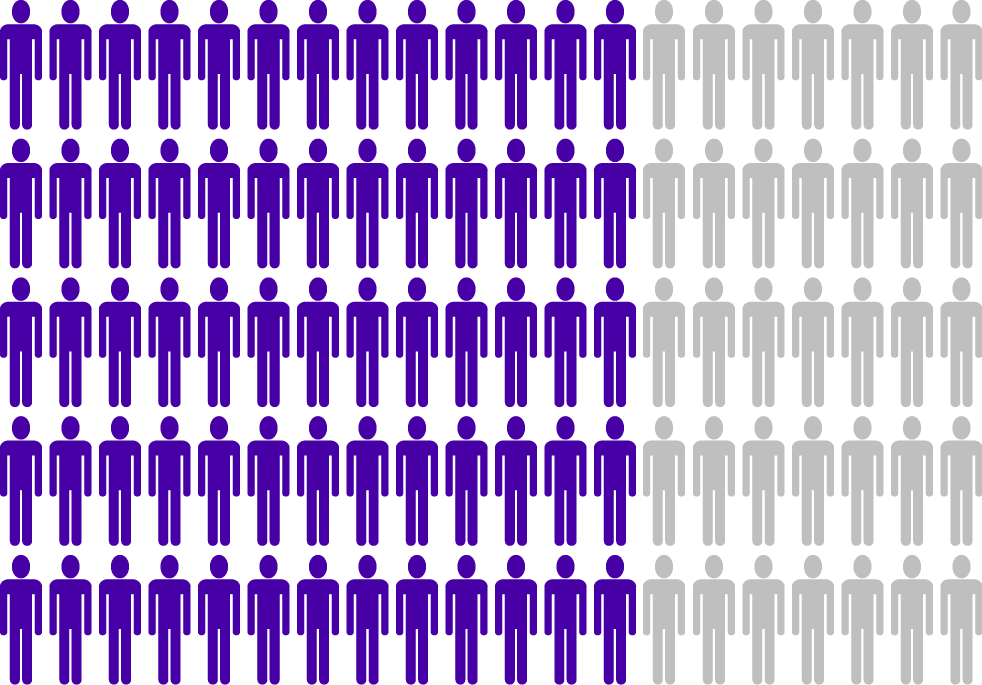


1. If each figure in the picture is 1 person who takes a treatment for SHPT, for how many people would the treatment keep all 3 lab values within their recommended ranges?

- 90 out of 100 (90%)
- 80 out of 100 (80%)
- 65 out of 100 (65%)
- 20 out of 100 (20%)

Remember that each figure in the picture represents someone who takes a medicine or has an operation to treat SHPT. There are 100 figures in the picture. The treatment **would** keep all 3 lab values within the recommended ranges for the 65 figures shown in color. Therefore, 65 out of 100 people (65%) is the answer.

Treatment Feature: Chance That the Treatment Relieves Your SHPT Symptoms

SHPT treatments can help reduce some SHPT symptoms such as dry or itchy skin; pain in your muscles, joints, or bones; joint stiffness; and muscle weakness. Relieving these symptoms will help make you feel better.

If you have an operation to remove 1 or more of your parathyroid glands and the treatment relieves your SHPT symptoms, then you will not need additional treatment.

If you take an SHPT medicine and the treatment relieves your SHPT symptoms, you will still need to continue taking the medicine.

Even if you still have symptoms after the treatment, the treatment may still be keeping your 3 lab values (PTH, phosphorus, and calcium) within the recommended ranges. Likewise, the treatment may relieve your symptoms, but that doesn’t always mean that the treatment is keeping your 3 lab values in the recommended ranges.

1. Which of the following SHPT symptoms would bother you the most?
   *(Check only 1)*

- Dry or itchy skin
- Pain in muscles, joints, and/or bones
- Joint stiffness
- Muscle weakness

1. Suppose your doctor tells you that you have SHPT and that you need to choose to either take an SHPT medicine or have an operation (parathyroidectomy).

- If you take Medicine A, there is a 60% chance that the medicine will keep your lab values in the recommended ranges and a 75% chance that the medicine will relieve your SHPT symptoms.
- If you take Medicine B, there is an 80% chance that the medicine will keep your lab values in the recommended ranges and a 5% chance that the medicine will relieve your SHPT symptoms.
- If you have the operation, there is a 60% chance that your lab values will be in the recommended ranges and a 35% chance that your SHPT symptoms will be relieved.

Please indicate which treatment you would choose.

| Treatment Feature | Medicine A | Medicine B | Operation |
| --- | --- | --- | --- |
| **Chance that the treatment keeps your 3 labs within their recommended ranges** | 60 out of 100 (60%) 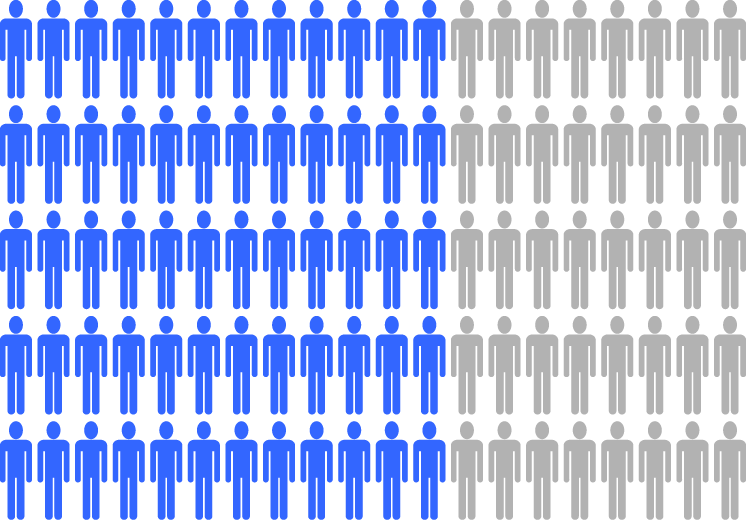 | 80 out of 100 (80%) 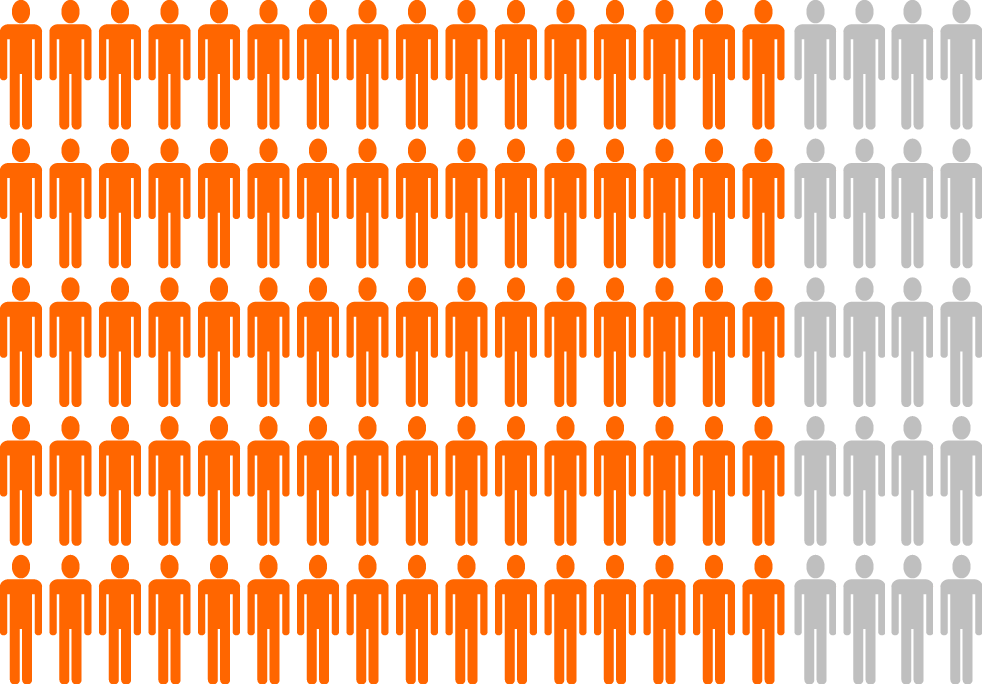 | 60 out of 100 (60%) 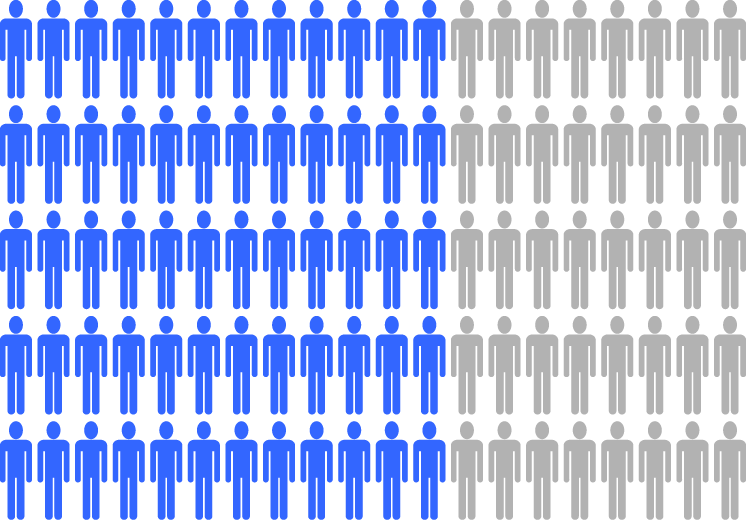 |
| **Chance that the treatment relieves your SHPT symptoms** | 75 out of 100 (75%) 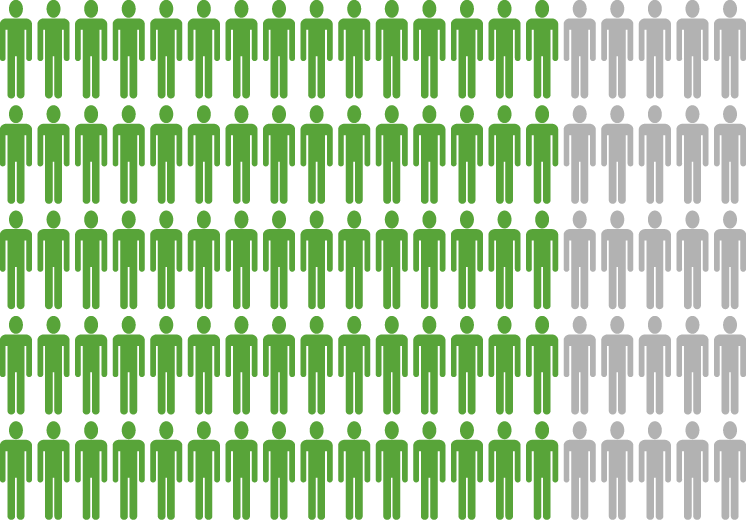 | 5 out of 100 (5%) 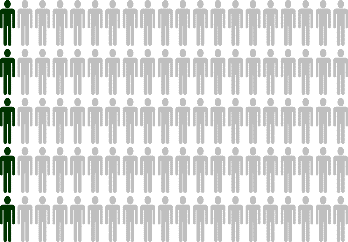 | 35 out of 100 (35%) 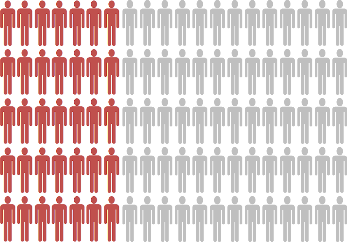 |
|  |  |  |  |
| **Which would you choose?** |  |  |  |

Treatment Feature: Risk of Low Blood Calcium

Some SHPT treatments may cause the levels of calcium in your bloodstream to be lower than normal.

If the calcium in your bloodstream gets too low, it can cause your heart to beat abnormally, which, in some cases, can lead to sudden death.

Two out of 100 people (2%) who have low blood calcium will have a seizure or convulsions.

If you have an operation to remove 1 or more of your parathyroid glands from your neck and your blood calcium level gets too low, then you may need to be admitted to the hospital, often more than once, until your doctor can get your blood calcium back up to normal levels.

If you take an SHPT medicine and your blood calcium level gets too low, your doctor will tell you to stop taking the medicine or adjust the dose of your medicine until your blood calcium gets back up to normal levels. If your blood calcium level gets too low, you may need to be admitted to the hospital until the doctors can get your blood calcium back up to normal levels.

1. Have you ever had a low blood calcium lab result?

- Yes
- No
- Don’t know / not sure

1. Have you ever had a seizure or convulsions?

- Yes
- No
- Don’t know / not sure

Thinking About the Risk of Having Low Blood Calcium

We will use pictures to help you think about how many patients will have low blood calcium because of an SHPT treatment.

Each figure in the picture below represents 1 person who receives an SHPT treatment (an operation or a medicine). There are 100 figures in each picture. The figures shown in color indicate people who **will** have low blood calcium because of the treatment. The figures in gray indicate people who **will not** have low blood calcium because of the treatment.

In this example:

- The figures in color show that 4 people out of 100 (4%) who have an SHPT treatment **will** have low blood calcium.
- The gray figures show that 96 people out of 100 (96%) who have an SHPT treatment **will not** have low blood calcium.

When there are more figures in color, your risk of having low blood calcium because of the treatment is higher.

Example 1:


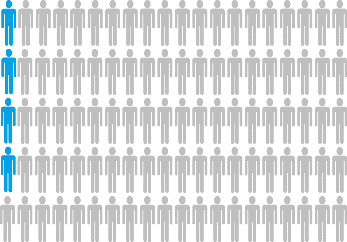


**There are 4 figures in color. That means that 4 people out of 100 (4%) who take this medicine will have low blood calcium because of the treatment.**

Please look at the picture below:


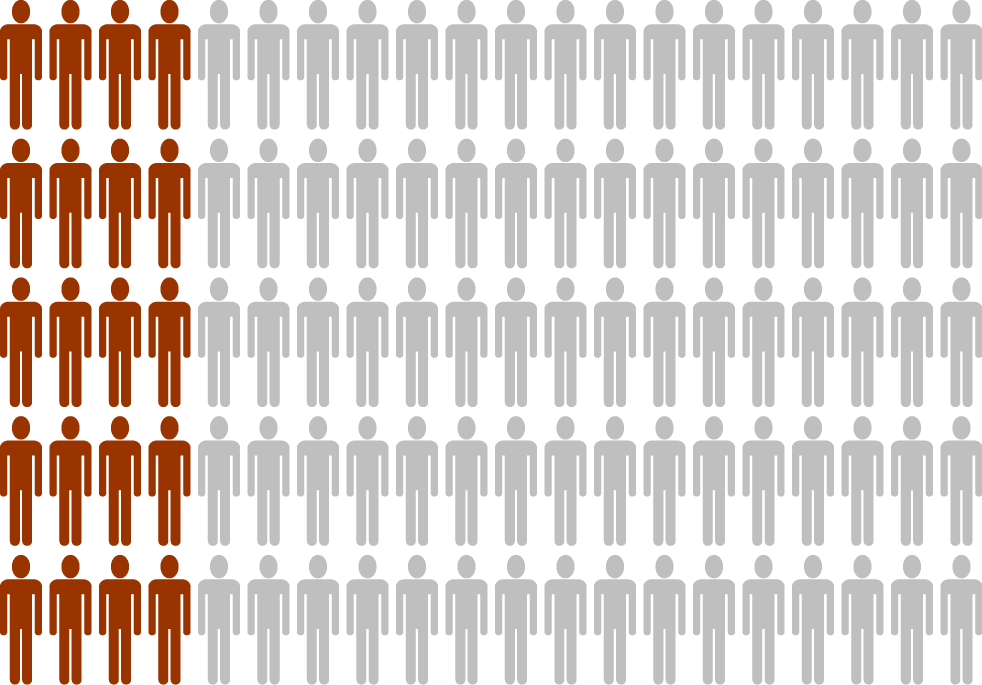


1. If each figure in the picture above is 1 person who takes an SHPT treatment, how many people who take the medicine will have low blood calcium?

- 10 out of 100 (10%)
- 20 out of 100 (20%)
- 40 out of 100 (40%)
- 60 out of 100 (60%)

Remember that each figure in the picture represents someone who has an operation for SHPT or who is taking an SHPT medicine. There are 100 figures in the picture. The figures in color indicate that 20 people **will** have low blood calcium because of the treatment. Therefore, 20 out of 100 people (20%) is the answer.

**Treatment Feature: Nausea and Vomiting**

Some medicines for SHPT may cause nausea and vomiting. We will ask you to think about 3 possible levels of nausea and vomiting:

- None
- Mild: nausea plus vomiting 1 day per week
- Moderate: nausea plus vomiting 2 to 3 days per week

1. Using the descriptions above, how would you describe the worst case of nausea and vomiting that you have ever experienced at any time in your life?

- None
- Mild: nausea plus vomiting 1 day per week
- Moderate: nausea plus vomiting 2 to 3 days per week
- Severe: nausea plus vomiting more than 2 to 3 days per week

Thinking About SHPT Treatments

1. Again, suppose your doctor tells you that you have SHPT and that you need to choose either to take an SHPT medicine or have an operation (parathyroidectomy).

| Treatment Feature | Medicine A | Medicine B | Operation |
| --- | --- | --- | --- |
| **Chance that the treatment keeps your 3 labs within their recommended ranges** | 60 out of 100 (60%) 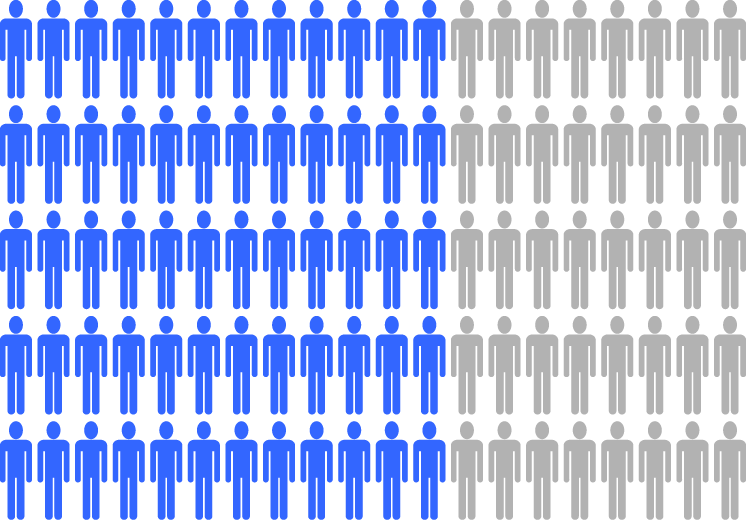 | 80 out of 100 (80%) 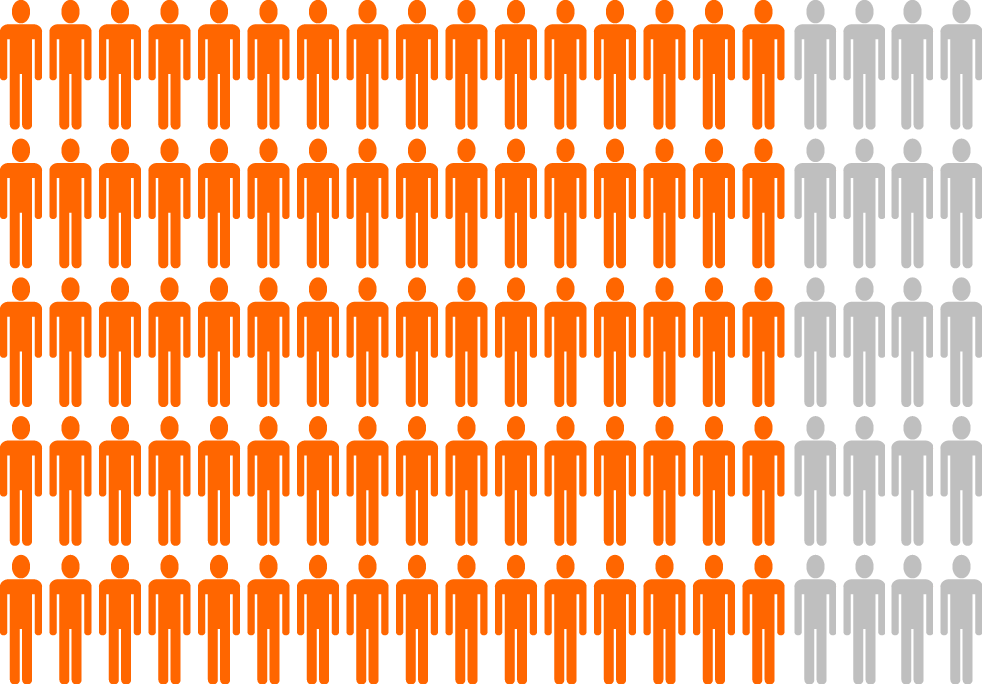 | 60 out of 100 (60%) 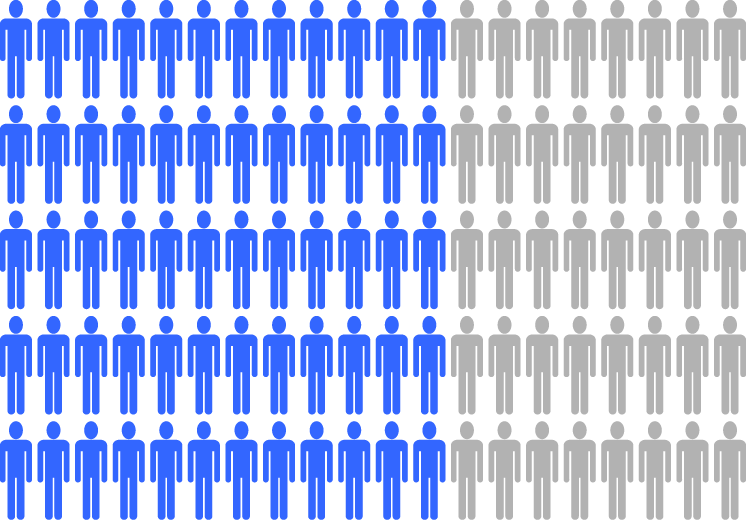 |
| **Chance that the treatment relieves your SHPT symptoms** | 75 out of 100 (75%) 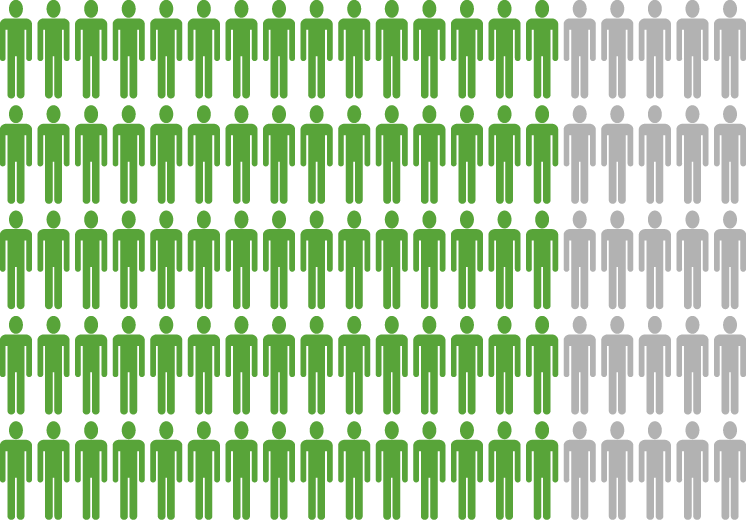 | 5 out of 100 (5%) 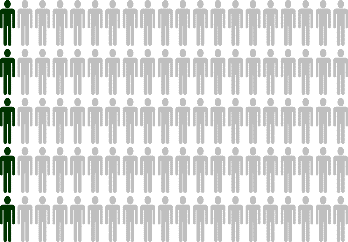 | 35 out of 100 (35%) 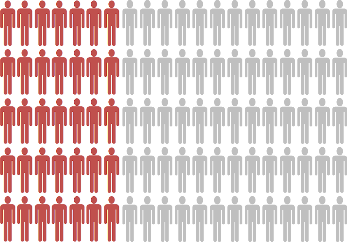 |
| **Risk of having low blood calcium** | 10 out of 100 (10%) 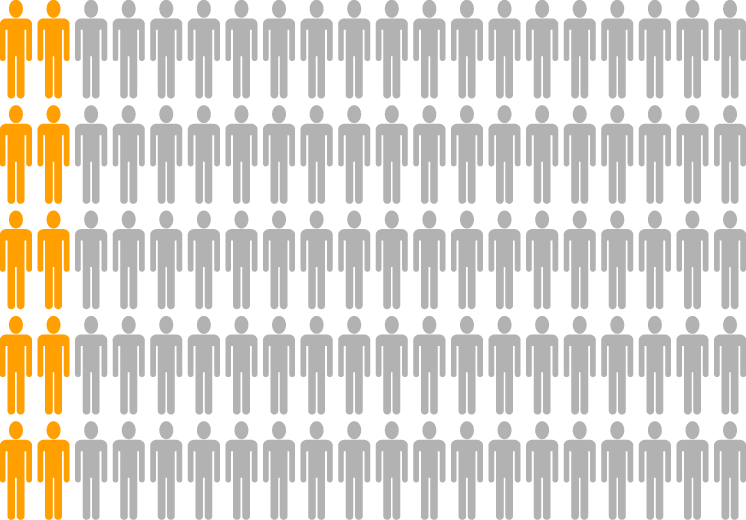 | 2 out of 100 (2%) 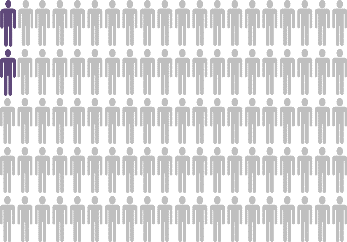 | 10 out of 100 (10%) 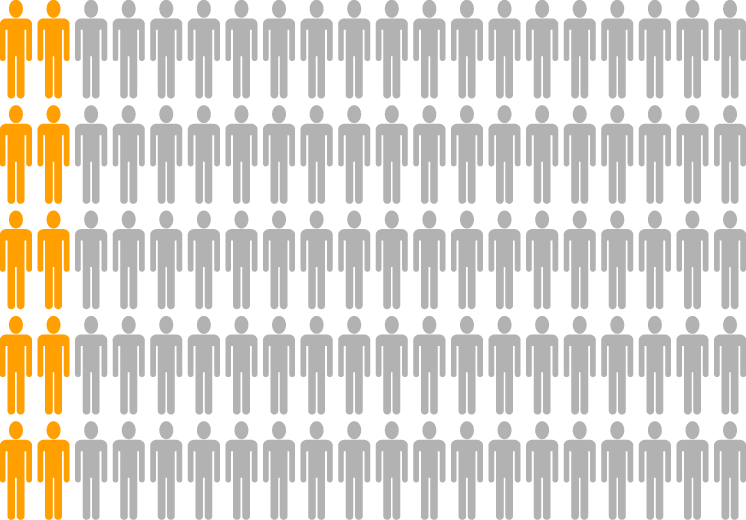 |
| **Nausea and vomiting** | Mild | None |  |
|  |  |  |  |
| **Which would you choose?** |  |  |  |

Medicine Feature: How You Receive the Medicine

We will ask you to think about 3 different ways you can receive SHPT medicines.

You can take oral medicines (pills) once a day. You could also take oral medicines (pills) once a week. If you take the medicine as a pill, you should take the medicine with food or shortly after a meal and at least 12 hours before you have a PTH blood test.

You could also receive a medicine by having a dialysis nurse give you the medicine through the dialysis line while you are receiving dialysis.

1. Have you ever received a medicine (any medicine) through an injection into the dialysis line during dialysis treatment?

- Yes
- No
- Don’t know / not sure

Medicine Feature: Out-of-Pocket Cost of Treatment

We will ask you to think about the costs of SHPT treatments. Treatment costs refer to what you personally would pay out of your own pocket, not what your health plan or insurance company would pay. For medicines, you would need to pay the cost of the medicine each month. For an operation, you would only pay the cost 1 time.

1. About how much do you personally pay for your SHPT medicine each month?

- Nothing
- $50 or less
- $51 to $100
- $101 to $250
- $251 to $500
- More than $500
- Don’t know / not sure
- I do not take an SHPT medicine

1. About how much do you personally pay for all your prescription medicines each month?

- Nothing
- $50 or less
- $51 to $100
- $101 to $250
- $251 to $500
- More than $500
- Don’t know / not sure

Thinking About SHPT Medicines

1. Again, suppose your doctor tells you that you have SHPT and that you need to choose to either take an SHPT medicine or have an operation (parathyroidectomy).

| Treatment Feature | Medicine A | Medicine B | Operation |
| --- | --- | --- | --- |
| **Chance that the treatment keeps your 3 labs within their recommended ranges** | 60 out of 100 (60%) 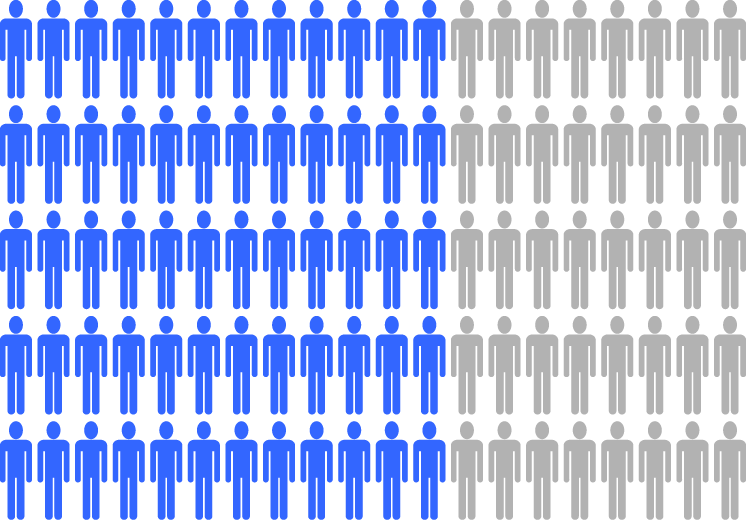 | 80 out of 100 (80%) 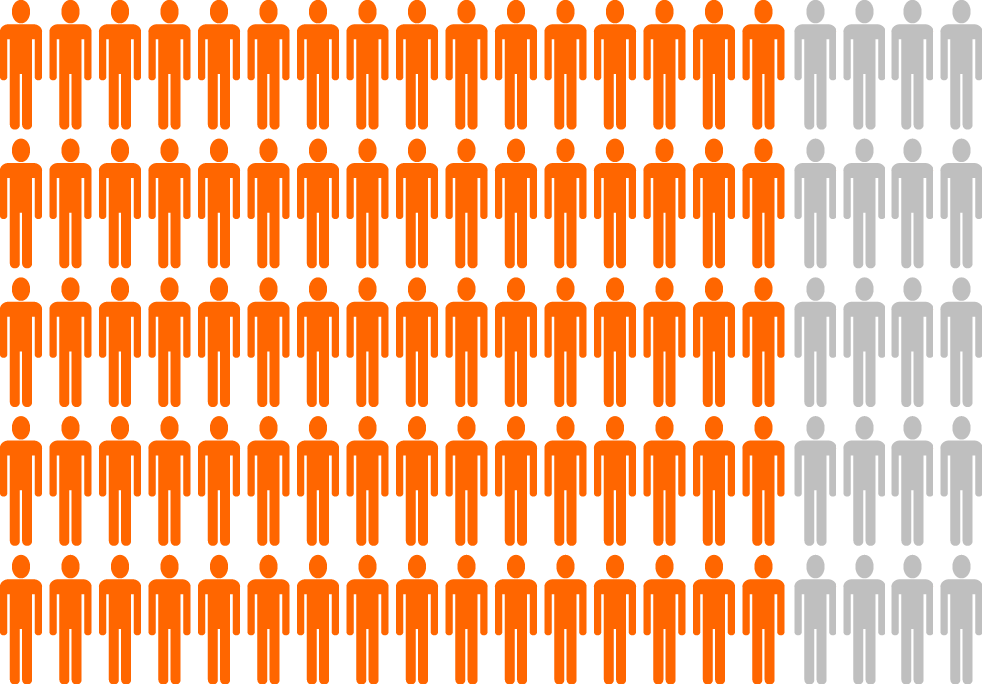 | 60 out of 100 (60%) 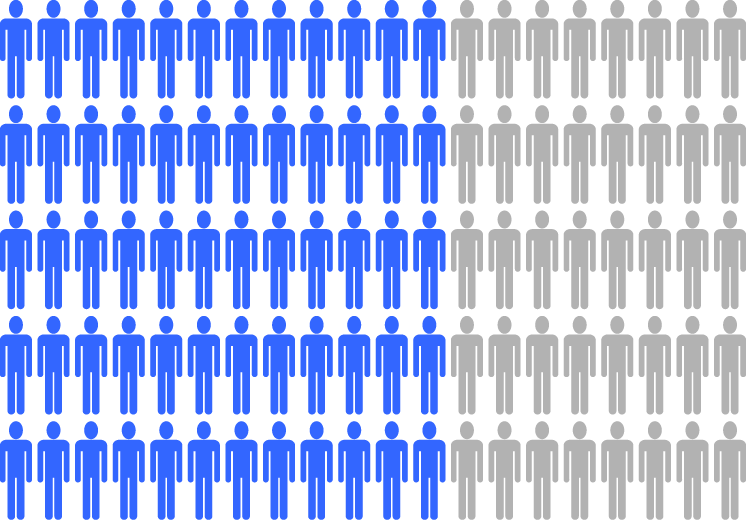 |
| **Chance that the treatment relieves your SHPT symptoms** | 75 out of 100 (75%) 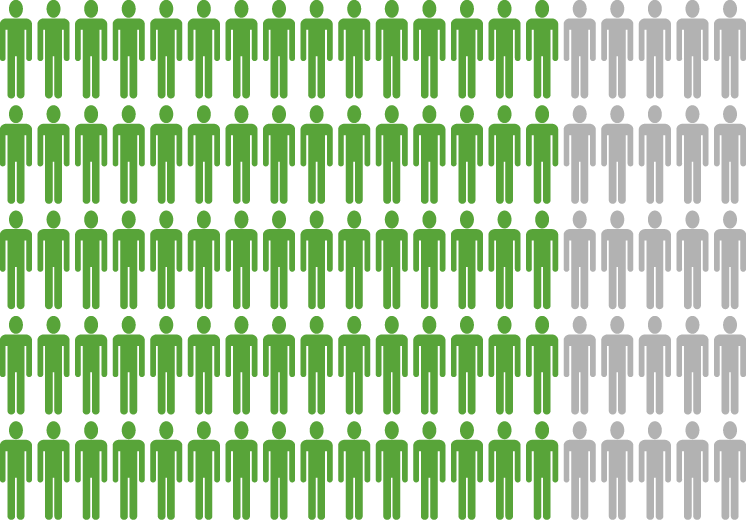 | 5 out of 100 (5%) 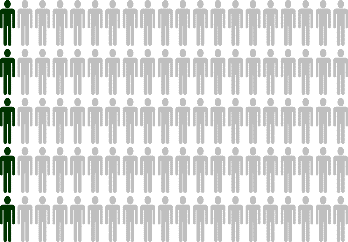 | 35 out of 100 (35%) 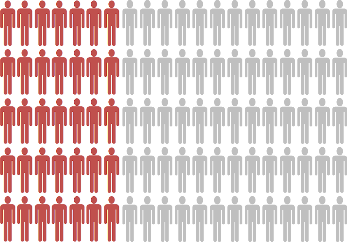 |
| **Risk of low blood calcium** | 10 out of 100 (10%) 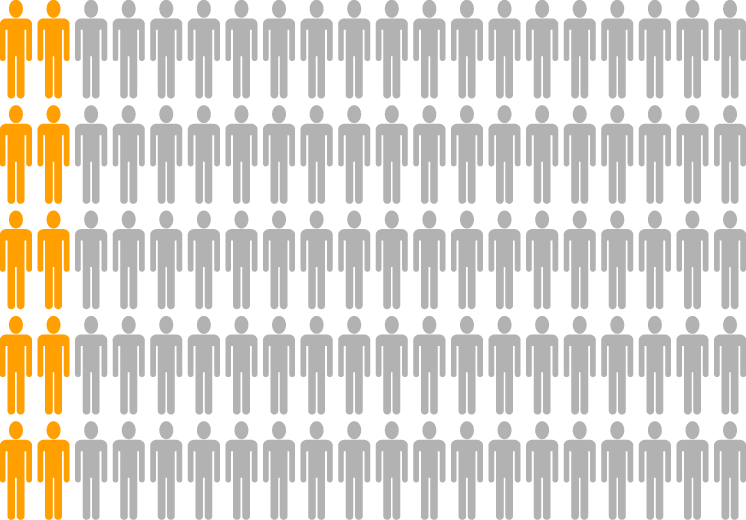 | 2 out of 100 (2%) 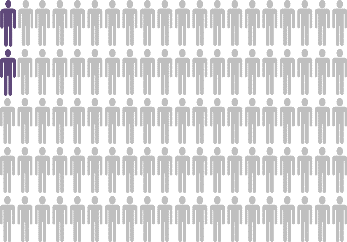 | 10 out of 100 (10%) 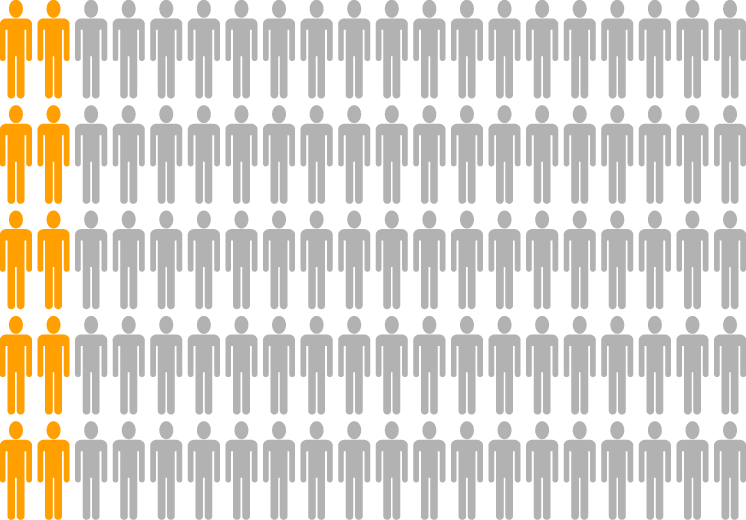 |
| **Nausea and vomiting** | Mild | None |  |
| **How you receive the medicine** | Pill once a day | Given through dialysis line during your regular dialysis treatment |  |
| **Out-of-pocket cost of treatment** | $100 each month | $50 each month | $500 one time (operation only) |
|  |  |  |  |
| **Which would you choose?** |  |  |  |

Your Opinions About SHPT Treatments

In each of the next 8 questions, we will show you different sets of possible SHPT treatments (2 possible medicines and an operation) your doctor might suggest. For each set of treatments, please choose the treatment you most prefer. There are no right or wrong answers.

PLEASE HELP US

Before you tell us which treatment you prefer in the following questions, we need your help with a problem we have in studies like this one. People often do not think much about the costs shown in the survey because they do not really have to pay the costs. They do not think about whether the features of an SHPT treatment would be worth the cost.

For example, if the costs are $50, $100, and $500, people often think of them as just "low," "medium," and "high." They do not really think about what they would have to give up—such as a restaurant meal or some new clothes—if they paid the costs.

The results of this study will not be used to set cost levels but will help us understand the value of possible SHPT treatments. If you do not pay attention to the costs in the questions, our results will be wrong. We will not get a true measure of how important the medicine features are.

Please help us understand your opinions by paying close attention to the cost of the treatment shown before deciding which treatment you prefer.

| Treatment Feature | Medicine A | Medicine B | Operation |
| --- | --- | --- | --- |
| **Chance that the treatment keeps your 3 labs within their recommended ranges** | 80 out of 100 (80%) 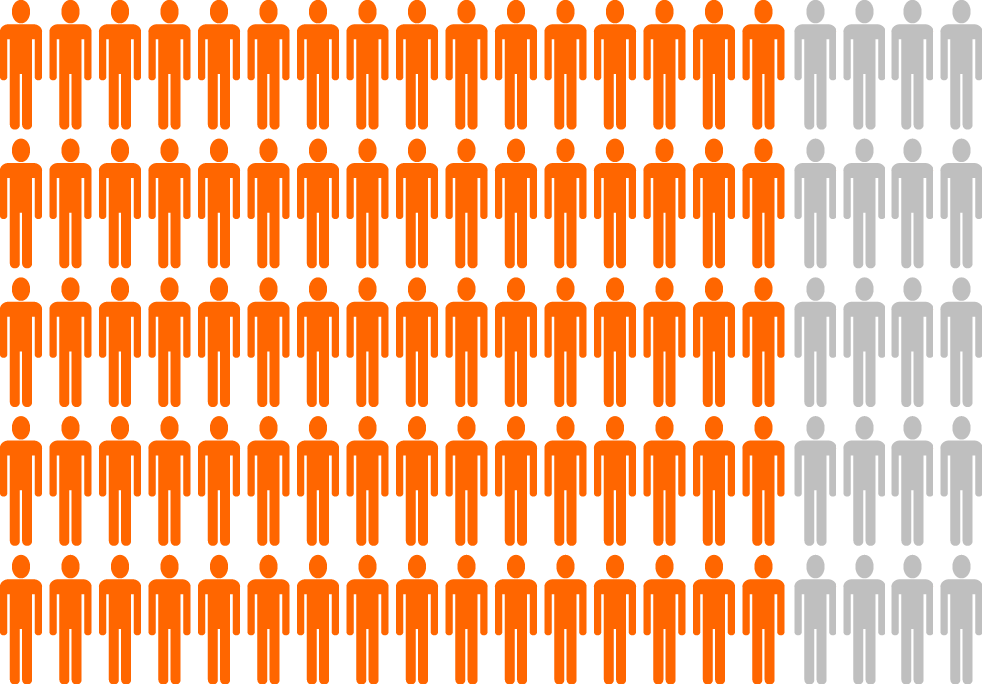 | 60 out of 100 (60%) 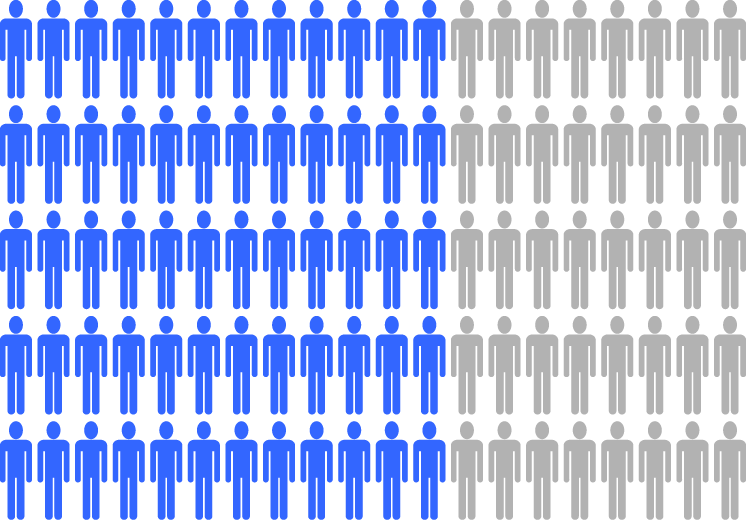 | 60 out of 100 (60%) 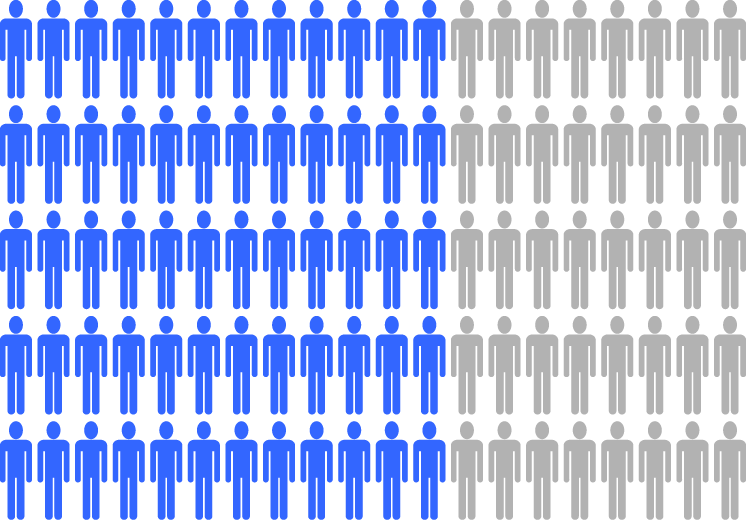 |
| **Chance that the treatment relieves your SHPT symptoms** | 35 out of 100 (35%) 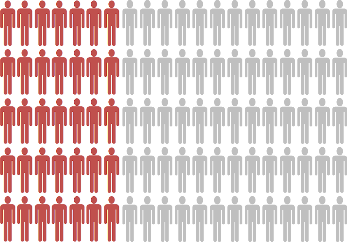 | 5 out of 100 (5%) 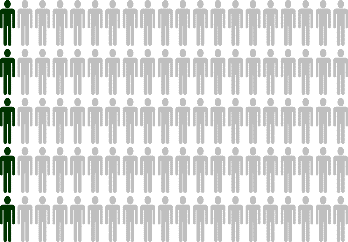 | 75 out of 100 (75%) 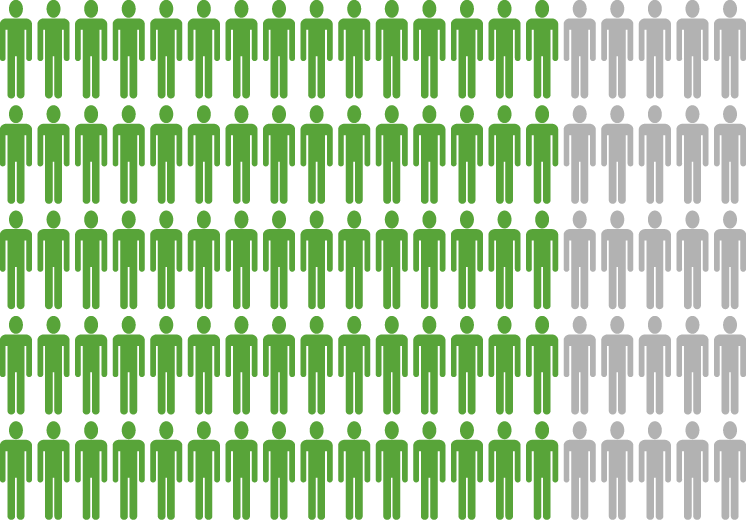 |
| **Risk of having low blood calcium** | 2 out of 100 (2%) 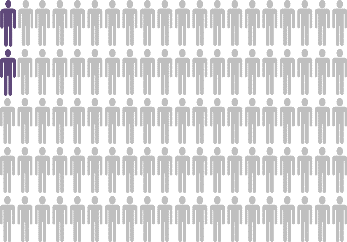 | 2 out of 100 (2%) 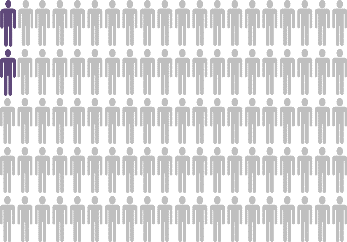 | 2 out of 100 (2%) 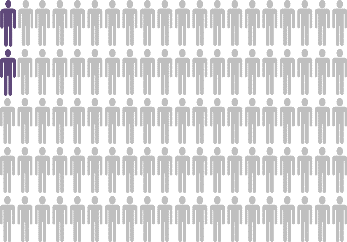 |
| **Nausea and vomiting** | Mild | Moderate |  |
| **How you receive the medicine** | Pill once a week | Pill once a week |  |
| **Out-of-pocket cost of treatment** | $100 each month | $100 each month | $500 one time (operation only) |
|  |  |  |  |
| **Which would you choose?** |  |  |  |

Other Features of SHPT Treatments

There are many features of SHPT treatments (including an operation and medicines) that were not included in the questions you just answered.

We are interested in knowing how bothered you would be by each of these possible features of SHPT treatments.

The table below shows 5 possible features of SHPT treatments. Please tell us which one would bother you the most. In other words, which one would you want to avoid the most?

| I would be  bothered by this  the MOST (Check only ONE) | Treatment Features |
| --- | --- |
|  | Being under general anesthesia during the operation |
|  | 10 out of 100 (10%) risk of having low blood calcium |
|  | Having a cut from 1 to 2 inches long on your neck |
|  | 1 out of 100 (1%) risk of damage to the nerves that control the vocal cords because of the operation |
|  | 1 out of 300 (0.3%) risk of serious bleeding during the operation |

Now, please tell us which of these same 5 features bothers you the least. In other words, which one would you choose if you had to experience one of these features?

| Treatment Features | I would be  bothered by this  the LEAST (Check only ONE) |
| --- | --- |
| Being under general anesthesia during the operation |  |
| 10 out of 100 (10%) risk of having low blood calcium |  |
| Having a cut from 1 to 2 inches long on your neck |  |
| 1 out of 100 (1%) risk of damage to the nerves that control the vocal cords because of the operation |  |
| 1 out of 300 (0.3%) risk of serious bleeding during the operation |  |

For the next 5 questions, we will show you a set of 5 features. For each set of features, please select the feature that would bother you the most by checking the box to the left of the feature.

Then, please select the feature that would bother you the least by checking the box to the right of that feature.

Please choose only 1 feature as the most bothersome and 1 feature as the least bothersome.

| I would be  bothered  by this  the MOST (Check only ONE) | Treatment Features | I would be  bothered by this  the LEAST (Check only ONE) |
| --- | --- | --- |
|  | Needing to take a pill every day |  |
|  | Needing to stay in the hospital overnight after the operation |  |
|  | Having a cut from 1 to 2 inches long on your neck |  |
|  | Being under general anesthesia during the operation |  |
|  | 2 out of 100 (2%) risk of having a seizure or convulsions |  |

| I would be  bothered by this  the MOST (Check only ONE) | Treatment Features | I would be  bothered by this  the LEAST (Check only ONE) |
| --- | --- | --- |
|  | Needing to stay in the hospital overnight after the operation |  |
|  | 1 out of 100 (1%) risk of dying because of the operation |  |
|  | 5 out of 100 (5%) risk of having a hoarse voice for up to 6 months after the operation |  |
|  | Being under general anesthesia during the operation |  |
|  | 10 out of 100 (10%) risk of having low blood calcium |  |


| I would be  bothered by this  the MOST (Check only ONE) | Treatment Features | I would be  bothered by this  the LEAST (Check only ONE) |
| --- | --- | --- |
|  | Needing to stay in the hospital overnight after the operation |  |
|  | 1 out of 100 (1%) risk of damage to the nerves that control the vocal cords because of the operation |  |
|  | 1 out of 300 (0.3%) risk of serious bleeding during the operation |  |
|  | 5 out of 100 (5%) risk of having a hoarse voice for up to 6 months after the operation |  |
|  | Needing to take a pill every day |  |

| I would be  bothered  by this  the MOST (Check only ONE) | Treatment Features | I would be  bothered by this  the LEAST (Check only ONE) |
| --- | --- | --- |
|  | 1 out of 100 (1%) risk of dying because of the operation |  |
|  | 2 out of 100 (2%) risk of having a seizure or convulsions |  |
|  | Having a cut from 1 to 2 inches long on your neck |  |
|  | 5 out of 100 (5%) risk of having a hoarse voice for up to 6 months after the operation |  |
|  | 1 out of 300 (0.3%) risk of serious bleeding during the operation |  |

| I would be  bothered  by this  the MOST (Check only ONE) | Treatment Features | I would be  bothered by this  the LEAST (Check only ONE) |
| --- | --- | --- |
|  | 2 out of 100 (2%) risk of having a seizure or convulsions |  |
|  | 10 out of 100 (10%) risk of having low blood calcium |  |
|  | Needing to take a pill every day |  |
|  | 1 out of 100 (1%) risk of dying because of the operation |  |
|  | 1 out of 100 (1%) risk of damage to the nerves that control the vocal cords because of the operation |  |

1. If all SHPT medicines worked equally well, how would you choose to receive the medicine? (check only one answer)
   - Pill once a day
   - Pill once a week
   - Given through dialysis line during your regular dialysis treatment
2. Please describe the main reason why you would prefer receiving your SHPT medicine through the dialysis line during your regular dialysis treatment to taking a pill once a day:

______________________

Other Questions About You

1. In what year were you born? ___________
2. What is your gender?

- Female
- Male
- Prefer not to answer

1. How would you describe your race or ethnicity? (C*heck all that apply*)

- White or Caucasian
- Black or African American
- Asian
- Hispanic or Latino
- Native Hawaiian or Other Pacific Islander
- American Indian or Alaska Native
- Other (please specify) ____________________
- Prefer not to answer

1. What is your marital status?

- Single / never married
- Married / living as married / civil partnership
- Divorced or separated
- Widowed / surviving partner
- Other
- Prefer not to answer

1. What is the highest level of education you have completed?

- Less than high school
- Some high school
- High school or equivalent (e.g., GED)
- Some college but no degree
- Technical school
- Associate’s degree (2-year college degree)
- 4-year college degree (e.g., BA, BS)
- Some graduate school but no degree
- Graduate or professional degree (e.g., MBA, MS, MD, PhD)
- Prefer not to answer

1. Which of the following best describes your employment status?

- Employed full-time
- Employed part-time
- Homemaker
- Student
- Retired
- Disabled / unable to work
- On medical leave of absence from work
- Unemployed but looking for work
- Unemployed and not looking for work
- Prefer not to answer

1. What type of health insurance do you have? (*Check all that apply.*)

- I do not have health insurance
- Private insurance that I pay for myself
- Private insurance that my or my spouse’s employer pays all or part of
- Medicaid
- Medicare
- Veterans Health insurance
- Other
- Don’t know / not sure
- Prefer not to answer

1. What was your total household income before tax and other deductions in 2014?

- Less than $20,000
- $20,000 to $29,999
- $30,000 to $39,999
- $40,000 to $49,999
- $50,000 to $59,999
- $60,000 to $69,999
- $70,000 to $79,999
- $80,000 to $89,999
- $90,000 to $99,999
- $100,000 to $149,999
- $150,000 to $199,999
- $200,000 or more
- Don’t know / not sure
- Prefer not to answer
